# Medical Opinion and Movement

**Published:** 1907-03-30

**Authors:** 


### Medical Opinion and Movement.

With the death of M. Berthelot, France has lost one of her ablest sons of science of the last century. Overcome with grief at the loss of his wife after a severe and protracted illness, he passed away within a few hours of his wife's death on March 18. He was 80 years of age. His contributions to chemical knowledge gained for him a world-wide reputation. It may justly be claimed for him that he laid the foundation-stones of organic synthetic chemistry, and we owe almost entirely to his genius for research our knowledge of the chemical constitution of glycerine and the fats. In spite of his arduous and extended scientific work, he found time to fulfil important functions of State. In 1881 he was elected a life Senator. In 1885 he was appointed Minister of Public Instruction, and in 1895 he was Minister of Foreign Affairs. His whole life forms a splendid example to his countrymen and to the whole world of strenuous and fruitful work in the pursuit of knowledge and for the benefit of mankind.

A Conference on the Teaching of Hygiene and Temperance in the Universities and Schools of the British Empire is to be convened on St. George's Day, April 23, during the visit of the Colonial Premiers to this country. This Conference has been arranged by a committee of leading medical men and others, who have specially interested themselves in this question, numbering among them Sir Lauder Brunton, Sir Thomas Barlow, Sir Victor and Lady Horsley, and Professor Sims Woodhead. Lord Elgin has signified his approval of the Conference, and has promised to attend if his engagements at the Colonial Conference permit. Lord Strathcona and Mount Royal, G.C.M.G., and Sir John Gorst, K.C., will respectively take the chair at the morning and afternoon sessions. The Colonial systems of teaching these subjects will be described by educational experts and papers will also be read by Continental savants. Further information and tickets of admission may be obtained from the Honorary Secretary, Miss St. John Wileman, 11 Chandos Street, Cavendish Square, W.

A new and successful treatment of leprosy is reported to have been discovered by Dr. Diesing, staff surgeon of the Imperial German Colonial troops, who was for many years in the Cameroons and later in South-West Africa. The treatment consists in the subcutaneous injection of iodoform emulsion. He uses an emulsion of olive oil, containing 30 per cent. iodoform, and injects from 2 to 8 cubic centimetres daily. He makes the injections at first in the neighbourhood of the diseased skin, and when these lesions have disappeared, in any part of the body most suitable, preferably the back or extremities. Other forms of iodine or iodoform administration were of no effect, but the author finds that the healing process is hastened by painting the leprous parts with tincture of iodine. No symptoms of iodoform poisoning showed themselves with the above-mentioned doses. The local effect of the injections appears in three or four days, but at the end of fourteen days' treatment leprous formations far removed from the actual point of injection begin to disappear. Dr. Diesing suggests that the efficacy of the treatment may be due either to a general disinfection of the system by the iodine set free from the emulsion *in statu nascendi*, or else that by killing the microbes at the seat of injection, antitoxins are set free which bring about the extinction of the leprous microbes throughout the body.

We understand that the New York State is endeavouring to ensure a supply of pure milk to the public by a Bill, introduced into the New York State Assembly for the pasteurisation of all milk sold in New York City. Pasteurising stations are to be established at which every quart of milk taken into the city must pass through the process of pasteurisation, and heavy penalties are enacted upon those convicted of dealing in or selling milk not so treated. In this way the public may secure a milk less harmful and freer from contamination than heretofore, but it may be seriously doubted whether such a measure of universal pasteurisation is a satisfactory solution to the problem of obtaining a pure milk supply. Professor V. Behring has recently dealt with the question in a speech at the Berlin meeting of the German Council of Agriculture, in which he compared the problem of a wholesome milk supply to that of a wholesome water supply. He pointed out that at one time none but germless water, sterilised by heat, was considered fit and hygienic for drinking. Now boiled water was regarded as unsatisfactory, and hygienic measures aimed rather at a proper supervision of the water source and of its transport to the consumer. He thought the same applied to milk. Tubercle poison is not rendered innocuous by boiling, and it is doubtful whether the virus of scarlet fever can be destroyed in this way. Scarlet fever infection should be prevented by supervision of those handling the milk, and measures should be taken to prevent the sale of milk from tuberculous cows. It is only by thorough measures of inspection and supervision both as to the source of the milk and the contamination to which it is liable at the different stages of its transit to the consumer that we can look for a satisfactory solution of this important problem.

